# Theory of mind profile and cerebellar alterations in remitted bipolar disorder 1 and 2: a comparison study

**DOI:** 10.3389/fnbeh.2022.971244

**Published:** 2022-09-08

**Authors:** Giusy Olivito, Michela Lupo, Libera Siciliano, Andrea Gragnani, Marco Saettoni, Corinna Pancheri, Matteo Panfili, Fabiana Pignatelli, Roberto Delle Chiaie, Maria Leggio

**Affiliations:** ^1^Ataxia Laboratory, Fondazione Santa Lucia IRCCS, Rome, Italy; ^2^Department of Psychology, Sapienza University of Rome, Rome, Italy; ^3^Servizio di Tutela della Salute Mentale e Riabilitazione dell’Età Evolutiva ASL, Rome, Italy; ^4^Scuola di Psicoterapia Cognitiva (SPC), Grosseto, Italy; ^5^Associazione Psicologia Cognitiva (APC)/Scuola di Psicoterapia Cognitiva (SPC), Rome, Italy; ^6^Unità Funzionale Salute Mentale Adulti ASL Toscana Nord-Ovest Valle del Serchio, Pisa, Italy; ^7^Department of Neuroscience and Mental Health–Policlinico Umberto I Hospital, Sapienza University of Rome, Rome, Italy

**Keywords:** social cognition, mentalizing, emotion, voxel-based morphometry, gray matter

## Abstract

The literature on social cognition abilities in bipolar disorder (BD) is controversial about the occurrence of theory of mind (ToM) alterations. In addition to other cerebral structures, such as the frontal and limbic areas, the processing of socially relevant stimuli has also been attributed to the cerebellum, which has been demonstrated to be involved in the above-mentioned disorder. Nevertheless, the cerebellar contribution to ToM deficits in bipolar patients needs to be elucidated further. To this aim, two tests assessing different components of ToM were used to evaluate the ability to appreciate affective and mental states of others in 17 individuals with a diagnosis of BD type 1 (BD1) and 13 with BD type 2 (BD2), both in the euthymic phase, compared to healthy matched controls. Cerebellar gray matter (GM) volumes were extracted and compared between BD1 and controls and BD2 and controls by using voxel-based morphometry. The results showed that BD1 patients were compromised in the cognitive and advanced components of ToM, while the BD2 ToM profile resulted in a more widespread compromise, also involving affective and automatic components. Both overlapping and differing areas of cerebellar GM reduction were found. The two groups of patients presented a pattern of GM reduction in cerebellar portions that are known to be involved in the affective and social domains, such as the vermis and Crus I and Crus II. Interestingly, in both BD1 and BD2, positive correlations were detected between lower ToM scores and decreased volumes in the cerebellum. Overall, BD2 patients showed a more compromised ToM profile and greater cerebellar impairment than BD1 patients. The different patterns of structural abnormalities may account for the different ToM performances evidenced, thus leading to divergent profiles between BD1 and BD2.

## Introduction

Bipolar disorder (BD) is a severe, chronic, and debilitating psychiatric disease characterized by episodes of mania (BD type I, BD1), or hypomania (BD type II, BD2), and depression (Merikangas et al., 2007; Prieto et al., 2014) with interepisode remission periods. Growing evidence has shown that patients with BD exhibit prominent cognitive impairments involving executive function, attention, verbal, and episodic memory (Bora et al., 2009; Bourne et al., 2013). Such impairments have been shown to persist during periods of clinical remission (Torres et al., 2007; Bora et al., 2011; Mann-Wrobel et al., 2011) and to correlate negatively with social and occupational adjustment (Martínez-Arán et al., 2004; Martino et al., 2008; Harvey et al., 2010). In addition to the neuropsychological aspects of BD, several studies have shown that social cognition (SC) deficits are also evident in BD both during the depressive and manic phases (Bora et al., 2005; Samamé et al., 2012; Bora and Pantelis, 2016). SC is a multidimensional psychological domain that involves a complex set of processes that enable adaptive social interaction, such as the representation of internal somatic states, knowledge about the self, perception of others, and interpersonal motivations (Amodio and Frith, 2006). It includes skills ranging from the recognition of basic emotions to the more complex processes of Theory of Mind (ToM), such as the ability to recognize and attribute mental states to others to explain and predict their behavior (Baron-Cohen et al., 1985). It includes both affective and cognitive components and implies the capacity to recognize the emotions, intentions, and thoughts (state of mind) of another person in or out of a social context (Meltzoff and Moore, 1989).

Available studies suggest that BD patients exhibit significant deficits in ToM (Kerr et al., 2003; Bora et al., 2005; Samamé et al., 2012). This ability allows us to reason about other people’s mental states and emotions (Fernyhough et al., 2008) and to decode nonverbal signals, such as those sent through the eyes (Adams et al., 2010). Most studies that have measured affective ToM with the test “Reading the Mind in the Eyes” (RMET; Baron-Cohen et al., 2001) in BD have found deficiencies in this population, in which bipolar participants scored significantly lower than the control group. While SC impairment has been described in the behavioral profile of BD (Bora et al., 2016) during both the depressive and manic phases, the current literature on the euthymic phase is discordant (Samamé et al., 2015; Aparicio et al., 2017). Indeed, the first study specifically designed to assess ToM in BD reported that both symptomatic manic and depressive patients had impairments, while patients in remission had a comparable performance to healthy controls (Kerr et al., 2003). In contrast, other studies in euthymic BD patients showed impairments in ToM tasks (Bora et al., 2005; Olley et al., 2005; Inoue et al., 2006; Lahera et al., 2008). Anatomically, ToM impairment in BD patients has been mainly related to structural and functional abnormalities in brain regions (i.e., the ventromedial pre-frontal cortex), which are crucial for social cognitive abilities (Bora et al., 2012; Delvecchio et al., 2013). However, among the numerous brain regions responsible for the processing of socially relevant stimuli, the cerebellum is acquiring an increasingly important role in light of the anatomical and functional connections found between this structure and the cortical and subcortical areas involved in emotional and social processing such as limbic areas (Schmahmann and Pandya, 1997) and specific portions of the frontal and temporoparietal lobes (Van Overwalle et al., 2014, 2019; Van Overwalle and Marien, 2016).

Although recognized as a structure primarily involved in motor control and coordination, a general consensus has been reached in recent decades on the role of the cerebellum in modulating cognitive function (Schmahmann and Sherman, 1998; Olivito et al., 2018, 2020) and, more recently, in the modulation of emotional response and mood (Lupo et al., 2019). Indeed, studies on patients with acquired cerebellar lesions or affected by degenerative cerebellar pathology have allowed us to identify alterations in more sophisticated aspects of human behavior, such as social behavior and personality (Schmahmann and Sherman, 1998; Lupo et al., 2018a, b; Clausi et al., 2019a). Among the behavioral alterations most commonly reported in these patients, changes in the emotional sphere are described, such as greater irritability, impulsivity, anxiety, dysphoria, emotional lability, difficulty in recognizing emotions, and attributing mental states of others, components that fall into the SC domain (Schmahmann and Sherman, 1998; Lupo et al., 2018a, b; Clausi et al., 2019b). On the other hand, several clinical studies have highlighted the presence of structural alterations of the cerebellum in subjects affected by various psychopathologies, such as schizophrenia (Okugawa et al., 2007), major depressive disorder (Bora and Berk, 2016), and bipolar disorder (Sani et al., 2016; Lupo et al., 2021), leading to the hypothesis that the cerebellum may be involved in the genesis of some typical symptoms of these disorders. Different studies aimed at analyzing the anatomical substrate of BD have found a significant pattern of atrophy involving cerebellar regions, such as the cerebellar vermis, the anterior lobule V and posterior lobules Crus I and Crus II (Mills et al., 2005; Lupo et al., 2021), which are known to be strictly connected to frontal, temporal, and limbic social brain regions. A recent resting-state fMRI study found that cerebello-cerebral functional connectivity alterations persist during the euthymic phase and differentiate BD1 and BD2 (Olivito et al., 2022). To our knowledge, the relation between the cerebellum and ToM in BD patients has never been specifically investigated. A previous whole-brain study found no relation between the cerebellar volume and ToM performance in a mixed population of BD patients, while increased cerebellar GM was found to be related to better performances at ToM tasks in healthy subjects (Quidé et al., 2020).

Considering these observations, in the present study, we aimed to characterize the ToM profile in BD1 and BD2 under clinical remission and to investigate the relation between cerebellar alterations and emotional and social functioning in BD1 and BD2 during the euthymic phase.

## Materials and Methods

### Participants

Seventeen patients with BD type 1 (mean age/SD:38.64/13.48; M/F:9/8) and 13 patients with BD type 2 (BD2) (mean age/SD, 41.42/14.38; M/F:6/7) were enrolled for this study. Both BD samples were used in previous studies from our group (Lupo et al., 2021; Olivito et al., 2022). Statistical analysis showed no significant differences between BD1 and BD2 in terms of age (*t test*: *t* = −1.39, *p* = 0.17) and sex distribution (chi-square: χ^2^ = 1.03, *p* = 0.31). Individuals with BD were recruited by an expert clinical psychiatrist from the Department of Psychiatry, Policlinico Umberto I Hospital. They all met the criteria for BD according to the Diagnostic and Statistical Manual of Mental Disorders, Fifth Edition (DSM-5) and were diagnosed using the Italian version of the Structured Clinical Interview for DSM-5—Clinician Version (SCID-5-CV; First et al., 2017). The following criteria were considered for BD patient inclusion: (i) aged between 18 and 60 years; (ii) first examination by a psychiatrist performed before age 40; (iii) euthymic mood for at least 3 months, and (iv) suitability for magnetic resonance imaging (MRI). For BD patient exclusion, the criteria included: (i) having other Axis-I psychiatric disorders; (ii) having intellectual disability; (iii) having a history of an organic brain disorder or neurological disorder; (iv) having any cerebral lesion on conventional MRI scans; (v) having a medical condition, such as cardiovascular disease or diabetes; (vi) having lifetime alcohol/substance abuse; and (vii) being pregnant. In accordance with the inclusion criteria, an expert clinical psychiatrist ensured that all the patients had been in the euthymic phase for at least 3 months by means of the Hamilton Depression Rating Scale (HDRS score <10; Hamilton, 1967) and Young Mania Rating Scale (YMRS score <12; Young et al., 1978). Additionally, an expert neurologist conducted a neurological evaluation for all BD patients, and the presence of cerebellar motor deficits was specifically assessed using the International Cooperative Ataxia Rating Scale (Trouillas et al., 1997), ranging from 0 (absence of a motor deficit) to 100 (presence of motor deficits at the highest degree). All BD1 and BD2 patients underwent the ToM assessment and MRI protocols. All patients were under medication but the dosages at the time of enrollement were not recorded.

Two specific control groups were enrolled for the ToM and MRI examinations. Forty healthy subjects (HS-ToM; mean age/SD, 41.1/12.3; M/F, 14/26) with no history of neurological or psychiatric illness were enrolled for the assessment of ToM. Statistical analysis showed no significant differences between BD1 and HS-ToM in terms of age (*t test*: *t* = 0.17, *p* = 0.86) and sex distribution (chi-square: χ^2^ = 0.00, *p* = 0.98) or between BD2 and HS-ToM (*t test* for age: *t* = −1.07, *p* = 0.29; chi-square for sex: χ^2^ = 1.45, *p* = 0.22). The Raven progressive matrices test (Raven, 1949) was administered in the three groups to verify the presence of an average intellectual level (>18.96). A second control group (HS-MRI) was used for the MRI analysis and was based on retrospective MRI data of healthy participants collected from 2014 to 2019 at the Neuroimaging Laboratory of Santa Lucia Foundation. HS-MRI was composed of 37 healthy subjects (mean age/SD = 46.8/14.2; M/F = 15/22) with no history of neurological or psychiatric illness. Again, statistical analysis revealed no significant differences between BD1 and HS-RM for age (*t test*: *t* = 1.35, *p* = 0.18) and sex distribution (chi-square: χ^2^ = 0.13, *p* = 0.71) or between BD2 and HS-RM (*t test* for age: *t* = 0.15, *p* = 0.87; chi-square for sex: χ^2^ = 0.69, *p* = 0.40).

The demographic characteristics and the scores obtained in the screening evaluation are reported in Table 1. Clinical details and current pharmacotherapy of BD1 and BD2 are reported in Table 2.

**Table 1:** Demographic data and clinical scores of the studied groups.

**Group**	**N.**	**Sex (M/F)**	**Age**	Raven’s 47*	ICARS**
BD1	17	9/8	38.6/13.4	28.7/2.7	1/1.5
BD2	13	6/7	41.2/14.3	27.7/7.1	1.1/2.6
HS-TOM	40	14/26	41.1/12.3	31.2/3.1	-

*Raven’s 47: cut-off = <18.96; **ICARS: International Cooperative Ataxia Rating Scale (Trouillas et al., 1997). The values are reported as mean and standard deviation (mean/SD).

**Table 2:** Details on BD1 and BD2 patients’ scores on HDRS and YMRS and current pharmacotherapy.

	**HDRS**	**YMRS**	**Antipsychotics**	**Lithium**	**Antiepileptics**	**Antidepressants**	**Anxiolytic**	**Polypharmacy**
	**(M/DS)**	**(M/DS)**	**(N)**	**(N)**	**(N)**	**(N)**	**(N)**	**(N)**
BD1	1.00/1.41	1.29/3.06	12	10	13	2	2	14
BD2	2.62/3.23	1.77/2.74	7	7	10	1	3	8

HDRS, Hamilton Depression Rating Scale; YMRS, Young Mania Rating Scale; the values are reported as mean and standard deviation (mean/SD).

### Theory of mind assessment

Two specific paper-and-pencil tests were used to evaluate and compare ToM skills between participants: the faux pas test (FP; Stone et al., 1998; Liverta Sempio et al., 2005) and the Italian version of the Reading the Mind in the Eyes test (RMET; Baron-Cohen et al., 2001; Serafin and Surian, 2004).

The FP test assesses a more complex and conscious component of ToM, since it evaluates the advanced human ability to infer others’ mental states by identifying false beliefs and improper affirmations that might impact others’ emotions. It consists of 20 short stories, 10 of which are targeted by the occurrence of a social “faux pas” (“faux pas” stories) and the other 10 by no occurrence of social “faux pas” (“no-faux pas” stories). Each story and certain related questions were read orally to participants while they had a duplicate of the story to read along and check back over. For each of the 20 stories, the participant was questioned whether anyone said anything inappropriate. When answered affirmatively, additional questions were asked to deepen participants’ understanding of mental and emotional states. Responses to “faux pas” stories were scored 1 if accurate and 0 if wrong, resulting in a maximum score of 6 for each story. The “no-faux pas” stories were scored two for each story if no faux-pas was detected properly. Two further control questions were asked for all 20 stories to confirm participants’ factual understanding of the stories and scored 1 or 0 (Stone et al., 1998). The “faux pas” detection question (question 1) together with the additional false belief questions asked when the faux pas was identified (questions 2–5) reflect the cognitive ToM component (i.e., “Did anyone say something they shouldn’t have said or something awkward?”), while the affective question (question 6; i.e., How do you think X felt?”) allows us to evaluate the affective ToM component.

The “Reading the Mind in the Eyes” test (RMET) is among the most commonly used tests for ToM evaluation and assesses the ability to identify mental states from gaze. Hence, it allows the evaluation of a more automatic component of ToM. Participants were required to recognize what male and female actors depicted in black and white photographs were feeling (e.g., desire, worry) or thinking (e.g., sceptical, thoughtful, etc.), by coupling one of four mental states with the expression conveyed by the eyes. The 36 items that constitute the RMET can be classified according to mental states’ valence as follows: eight positive stimuli (e.g., friendly), 12 negative stimuli (e.g., hostile), and 16 neutral stimuli (e.g., pensive; Hudson et al., 2020). Responses were scored 1 if accurate and 0 if wrong.

#### Data analyses

The distribution of variables was tested by the Shapiro-Wilk test (Oztuna et al., 2006). Since scores did not present a normal distribution across BD and HS-ToM groups (*p* < 0.05), a non-parametric statistics has been used. A Kruskal–Wallis test for multiple independent samples was performed for intergroup comparisons. The results were deemed statistically significant at *p* < 0.05. When significant differences were observed, pairwise comparisons were carried out using Dunn’s *post-hoc* test with Bonferroni correction for multiple testing. The statistical analyses were performed using Statistical Package for the Social Sciences (SPSS version 25).

### MRI acquisition protocol

BD1, BD2, and HS-RM underwent MRI scanning at 3T (Magnetom Allegra, Siemens, Erlangen, Germany) that included the acquisition of the following sequences: (1) dual-echo turbo spin echo (TSE; TR = 6,190 ms, TE = 12/109 ms); (2) fast-FLAIR (TR = 8,170 ms, 204TE = 96 ms, TI = 2,100 ms); and (3) 3D modified driven equilibrium Fourier transform (MDEFT) scans (TR = 1,338 ms, TE = 2.4 ms, matrix = 256 × 224 × 176, in-plane FOV = 250 × 250 mm^2^, slice thickness = 1 mm) to perform voxel-based morphometry analysis on cerebellar gray matter (GM) maps. The TSE scans of all patients were visually checked by an expert neuroradiologist to characterize the brain anatomy and inspect the presence of macroscopic structural abnormalities. Conventional MRI scans of HS-RM were revised to confirm the absence of any macroscopic abnormality in the brain according to the inclusion criteria.

### MRI data processing and analysis

The individual preprocessing of the cerebellum was performed by using the Spatially Unbiased Infratentorial Template (SUIT) toolbox (Diedrichsen et al., 2009) implemented in Statistical Parametric Mapping version 8 (Wellcome Department of Imaging Neuroscience; SPM-8^1^, accessed on 2 April 2009). The procedure included the following processing on each participant’s individual T1 anatomical images: the cerebellum was isolated, the isolated maps were hand-corrected if necessary, and each cropped image was normalized into SUIT space; the deformation parameters obtained by normalization were used to reslice the probabilistic cerebellar atlas into individual subjects’ space, and the images were smoothed using an 8-mm FWHM Gaussian kernel. Voxel-based morphometry was executed on cerebellar modulated GM maps entered into a voxelwise two-sample t test model as implemented in SPM-8 to distinctly compare the cerebellar GM volumes between the BD1 group and HS-MRI group and between the BD2 group and HS-MRI group. The analysis was restricted only to the voxels of the cerebellum by using an explicit exclusion mask. Age and sex were set as nuisance variables. The results were considered significant at *p* = < 0.05 after familywise error (FWE) cluster-level correction (clusters formed at *p* < 0.001 at uncorrected level).

### Correlations between ToM performance and cerebellar morphovolumetric parameters

Based on VBM analysis results, we performed a correlational analysis between the impaired ToM scores and the extracted lobular volumes of significantly reduced cerebellar GM areas in BD1 and BD2 compared to HS-MRI. The volume extraction was made using the FSL command line “fslstats” from the FMRIB software library (FSL^2^) applied to the modulated GM maps. The correlational analysis was performed by Spearman’s test by means of SPSS version 25.

## Results

### BD1 and BD2 ToM profile

The results of the Kruskal–Wallis test showed significant differences between the three groups in the total score of the “faux pas” stories (*H*_(2)_ = 19.576, *p* = 0.000), in the cognitive component scores (*H*_(2)_ = 20.592, *p* = 0.000), and in the affective component scores (*H*_(2)_ = 9.103, *p* = 0.011). Nonsignificant differences were detected in the total score of the “no-faux pas” stories (*H*_(2)_ = 0.690, *p* = 0.708). The pairwise comparisons, carried out by means of Dunn’s *post-hoc* test with Bonferroni correction for multiple testing, revealed that both the BD1 and BD2 groups had lower scores than healthy controls in the “faux pas” stories (BD1 vs. HS-TOM: *H* = 16.973, *p* = 0.010; BD2 vs. HS-TOM: *H* = 26.196, *p* = 0.000) and in the cognitive component (BD1 vs. HS-TOM: *H* = 17.232, *p* = 0.009; BD2 vs. HS-TOM: *H* = 26.979, *p* = 0.000). The pairwise comparisons for the affective component revealed a significantly worse performance of BD2 patients than healthy controls (BD2 vs. HS-TOM: *H* = 18.442, *p* = 0.014).

Nonsignificant differences were detected in the total score of the RMET among the three groups (*H*_(2)_ = 4.459, *p* = 0.108). Nonsignificant differences were identified for stimuli with either positive (*H*_(2)_ = 1.434, *p* = 0.448) or neutral (*H*_(2)_ = 3.984, *p* = 0.136) valence, while a significant difference was detected in response accuracy for stimuli with negative valence (*H*_(2)_ = 6.439, *p* = 0.040). The pairwise comparisons showed that BD2 patients were significantly less accurate than HS-TOM in detecting negative mental states (*H* = 15.895, *p* = 0.039).

**Figure 1:**
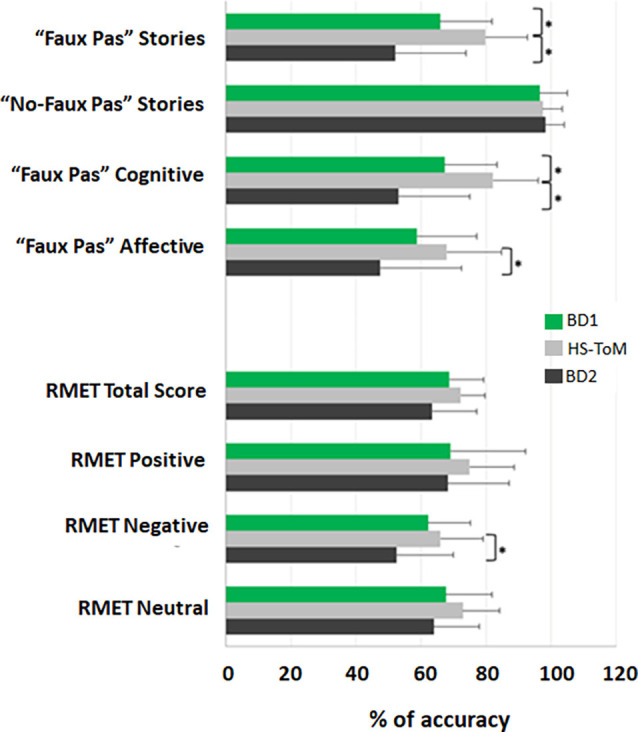
ToM profile of BD1 and BD2 patients. The results for each test are presented as the percentage of the total number of correct responses (accuracy); 0% indicates no correct answers, and 100% indicates totally correct. The mean and standard deviation of the accuracy are reported for BD1, HS-ToM, and BD2. *Statistical significance.

The mean and standard deviation of the scores obtained by each group in the ToM tests are reported in Table 3, while detailed statistics are reported in Table 4. The percentage accuracies, calculated as the percentage of correct responses on each test, are reported in Figure 1.

**Table 3:** Mean and Standard deviation of the ToM scores.

Group	**RMET**	**Positive**	**Negative**	**Neutral**	**“Faux Pas” Stories**	**“No-Faux Pas” Stories**	**Cognitive Component**	**Affective Component**
BD1	24.7/3.7	5.5/1.8	7.4/1.5	10.8/2.3	39.5/9.6	19.2/1.7	33.6/8	5.9/1.8
BD2	22.8/4.9	5.4/1.5	6.3/2.1	10.2/2.2	31.3/12.9	19.6/1.1	26.5/10.9	4.7/2.5
HS	26.0/2.7	6.0/1.1	7.9/1.6	11.6/1.8	47.9/7.7	19.5/1.2	41.1/6.9	6.8/1.7

The values are reported as mean and standard deviation (mean/SD) of scores in the RMET total (max = 36), in the stimuli with positive valence (max = 12), in the stimuli with negative valence (max = 8), in the stimuli with neutral valence (max = 16), in the “Faux Pas” Stories (max = 60), in the “No-Faux Pas” Stories (max = 20), in the Affective Component (max = 10), and in the Cognitive Component (max = 50).

**Table 4:** Results of statistical analysis between BD1, BD2, and HS in the ToM tests.

	**RMET**	**Positive**	**Negative**	**Neutral**	**“Faux Pas” Stories**	**“No-Faux Pas” Stories**	**Cognitive Component**	**Affective Component** [-8pt]
Kruskal-Wallis Test								
	0.108	0.488	0.040*	0.136	0.000*	0.708	0.000*	0.011*
Dunn’s *Post-Hoc*								
BD1 vs. HS	-	-	0.763	-	0.010**	-	0.009**	0.249
BD2 vs. HS	-	-	0.039**	-	0.000**	-	0.000**	0.014**
BD1 vs. BD2	-	-	0.643	-	0.667	-	0.591	0.756

*Results significant at *p* < 0.05. **Results significant at p-value adjusted using the Bonferroni correction for multiple comparisons.

### Cerebellar voxel-based morphometry

**Figure 2:**
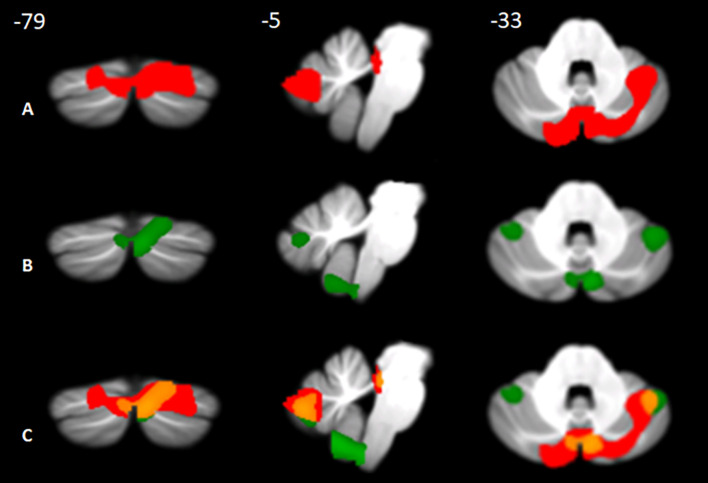
Between-group voxel-based comparison of cerebellar GM density. Cerebellar regions showing patterns of significantly reduced GM in BD1 **(A)** and BD2 **(B)** compared to MRI-HS are reported and superimposed on the Spatially Unbiased Infratentorial Template (SUIT; Diedrichsen et al., 2009) in coronal (y), sagittal (x), and axial (z) slices. The results are significant at p-values < 0.05 after FWE cluster-level correction. Images are shown in neurological convention. Regions of overlapping cerebellar GM loss **(C)** between BD1 (in red) and BD2 (in green) are reported in orange.

The results showed the presence of structural alterations in BD patients compared to the HS-MRI group at the level of the cerebellar hemispheres. Specifically, BD1 patients showed a pattern of reduced GM density both at the level of the anterior and posterior cerebellar portions with main involvement of the right hemisphere. A single large cluster of significant GM decrease was found with peak voxels in the right lobule V and extending to the right lobule I-IV, VI, crus I and crus II, the left crus I and II, and the vermis crus II, V, and VIIIA (Figure 2A). BD2 patients showed more diffuse cerebellar GM atrophy affecting both the left and right hemispheres. Several clusters of significant GM decrease were found involving the right lobule I-IV, V, VI, crus I, crus II, IX and VIIIb, the left VI, crus I, crus II, VIIb and IX, and the vermis crus II (Figure 2B). Detailed statistics and peak voxels showing the greatest significant differences in a cluster are reported in **Tables s 5A,B**. A pattern of overlapping cerebellar GM reduction in BD1 and BD2 was evident in the right lobule I-IV, V, Crus I and Crus II and the left Crus II and vermis Crus II (Figure 2C).

**Table 5:** Detailed statistics of voxelwise comparisons of cerebellar GM density (A: BD1 < HS-MRI; B: BD2 < HS-MRI).

	**Regions**	**Size**	**Side**	**Coordinates (mm)**			**Peak Z-scores**
				**x**	**y**	**z**	
**A)**	Lobule V	**26,837**	R	17	−42	−12	5.89
**B)**	Lobule V	**2,628**	R	17	−42	−12	5.81
	Lobule IX	**4,263**	R	−4	−60	−54	4.51
	Lobule VIIIb		L	−7	−43	−62	3.23
	Crus II	**5,675**	L	−43	−48	−46	4.20
	Crus I		L	−43	−43	−31	3.53
	Crus II	**3,596**	R	8	−78	−33	4.16
	Crus I		R	16	−79	−24	3.76
	Crus II		L	−5	−76	−31	3.47
	Crus I	**2,763**	R	45	−48	−30	3.94

Results are significant at *p* < 0.05 after FWE correction.

### Correlations between ToM scores and reduced GMVs

**Figure 3:**
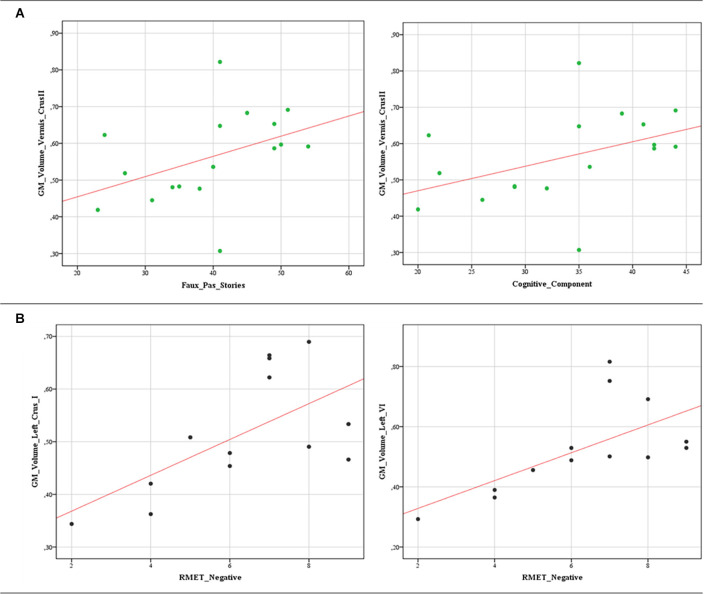
Data scatterplots. Significant correlations between decreased cerebellar GM volumes and the poor ToM scores in BD1 **(A)** and BD2 **(B)** patients are reported. **(A)** Positive correlations between the total score (*r* = 0.540; *p* = 0.025) and the cognitive component score (*r* = 0.511; *p* = 0.036) on the Faux Pas stories and GM volumes in the Vermis Crus II. **(B)** Positive correlations between the scores at the negative stimuli of the RMET and the reduced cerebellar GM volumes in the left crus I (*r* = 0.625; *p* = 0.022) and in the left lobule VI (*r* = 0.756; *p* = 0.003).

The correlational analysis performed by Spearman’s test revealed substantial correlations between significantly poor ToM scores and decreased GM volumes in both BD1 and BD2. Specifically, in BD1, both the total score and the cognitive component score on the Faux Pas stories were positively correlated with the GM volumes in the Vermis Crus II (“faux pas” stories: *r* = 0.540; *p* = 0.025; cognitive component: *r* = 0.511; *p* = 0.036). In BD2, significant positive correlations were detected between the scores at the negative stimuli of the RMET and the reduced cerebellar GM volumes in the left crus I (*r* = 0.625; *p* = 0.022) and in the left lobule VI (*r* = 0.756; *p* = 0.003). The data scatterplots of the significant correlations are shown in Figure 3.

## Discussion

To our knowledge, this is the first study differentiating BD1 and BD2 in terms of ToM profiles and cerebellar alteration patterns. In the present work, we tested the hypothesis that the cerebellum has a role in BD mentalizing deficits. Although available studies have shown that this clinical population exhibits significant deficits in social cognition both during the depressive and manic phases (Kerr et al., 2003; Bora et al., 2005, 2016; Samamé et al., 2012), research on the euthymic phase is still scant and somewhat discordant (Kerr et al., 2003; Samamé et al., 2015; Aparicio et al., 2017). From an anatomical point of view, BD is associated with structural and functional abnormalities in brain regions (i.e., the ventromedial pre- frontal cortex), which are important for social cognitive abilities (Bora et al., 2012; Delvecchio et al., 2013). Recently, the cerebellum has also been implicated in mentalizing processes, supported by its extensive connections with key social brain regions, such as limbic areas (Schmahmann and Pandya, 1997) and specific portions of the frontal and temporoparietal lobes (Van Overwalle et al., 2014, 2019; Van Overwalle and Marien, 2016). Intriguingly, clinical studies found ToM difficulties in patients affected by cerebellar pathology (Sokolovsky et al., 2010; D’Agata et al., 2011; Clausi et al., 2019a, 2021a,b), and neuroimaging data showed cerebellar activation during emotion recognition tasks (Habel et al., 2005). Interestingly, both structural and functional alterations of the cerebellum have been reported in patients affected by BD (Sani et al., 2016; Lupo et al., 2021; Olivito et al., 2022). According to these observations, the present study was conducted to compare the social-cognitive performance of euthymic BD1 and BD2 patients with that of healthy controls and to investigate structural cerebellar patterns that may be associated with possible social deficits in these populations.

Generally, our findings suggest that significant ToM deficits are present during the euthymic/remitted state of BD when compared to healthy controls. Interestingly, the studied cohorts showed different ToM profiles. When compared to healthy subjects, BD1 performed worse in the more advanced and conscious components of ToM, as assessed by the faux pas test, while the first automatic stage of ToM, as assessed by the RMET, was spared. Conversely, BD2 patients presented lower scores in both faux pas and RMET, suggesting that BD2 patients are more impaired than BD1 in ToM abilities. Indeed, BD2 patients were characterized by a lack of ability to “tune in” to the mental state of another person at both automatic and more conscious levels of ToM. Furthermore, BD2 patients exhibit a significant impairment in both Faux Pas cognitive and affective components.

Additionally, a significant pattern of cerebellar atrophy was found in BD patients, with more diffuse involvement of cerebellar structures in BD2 and a partial overlap between the two populations in terms of altered cerebellar portions. In particular, a pattern of common alterations was evident at the level of Crus II, a region known to be involved in specific aspects of mentalizing and higher-order emotional processes (Van Overwalle and Marien, 2016). We advance the hypothesis that the cerebellum is involved in mentalizing impairments observed in BD and that the social behavioral alterations described in BD1 and BD2 could be a consequence of the modulation action of specific cerebellar portions on the cortical network in which it acts. The strong link between the posterior cerebellum, i.e., Crus I and II, and key mentalizing areas in the cerebral cortex has been confirmed by several fMRI connectivity studies (Van Overwalle and Marien, 2016; Clausi et al., 2019a; Van Overwalle et al., 2020). In line with these observations, we speculate that the structural alteration of the cerebellar Crus I-II, known to be strictly related to more advanced ToM features (Van Overwalle and Marien, 2016; Sokolov, 2018), may structurally and functionally affect key mentalizing areas in the cerebral cortex and lead to mentalizing impairment observed in BD1 and BD2.

Interestingly, we also found that impaired ToM performance was correlated with GM volume in affected cerebellar regions. Within this framework, the specific patterns of correlations in BD1 and BD2 warrant further investigation. Specifically, BD1 impaired performances in the Faux Pas total scores and cognitive component correlated with reduced cerebellar GM in the vermis Crus II, while only BD2 scores at the negative stimuli of the RMET were correlated with the reduced cerebellar GM volumes in the left Crus I and in the left lobule VI.

Interestingly, in the study of Olivito et al. (2022), cerebello-cerebral functional connectivity (FC) changes have been investigated to differentiate BD1 and BD2 during interepisodic periods, and a different FC vulnerability has been found in BD2 patients compared to BD1 (Olivito et al., 2022). In the study by Olivito et al. (2022), the authors advanced the hypothesis that the different intensities and chronicity of the (hypo)manic symptoms in BD2 (Vieta and Suppes, 2008) may reflect different FC vulnerability or engage different mechanisms to maintain a state of euthymia. This is at least in part consistent with the present results. Indeed, we found that BD2 was more impaired both in terms of ToM profile and cerebellar atrophy pattern, with more diffuse involvement of anterior and posterior cerebellar areas. It must also be considered that while BD1 patients presented only with an altered cognitive component of ToM (cognitive Faux-Pas), BD2 patients were also impaired in affective ToM (Affective Faux Pas). While cognitive ToM concerns cognitive beliefs and reading the content of people’s minds, affective ToM concerns emotional states and functions involving affective influence, such as empathy, and refers to the ability to understand facial emotions that express feelings (Hein and Singer, 2008). This is consistent with the evidence that BD2 patients, but not BD1 patients, failed to recognize negative emotional stimuli as assessed by the RMET. A meta-analysis of neuroimaging studies has shown a variety of regions to be active during “emotional” vs. “neutral” experiences, including the midline regions in the vermis and with extension to lateral cerebellar crus I and lobule VI (Stoodley and Schmahmann, 2009). Consistently, in the present study, BD2 lower scores at RMET were linked to GM alterations in these cerebellar regions.

Although scarce, previous social cognition research in BD patients has highlighted the presence of social cognition deficits. In particular, the results of a large meta-analysis showed that BD patients exhibit deficits in several aspects of social cognition, with a large effect size for emotion recognition, moderate to large effect size for ToM, and a small to moderate effect size for social judgement and decision-making (Bora and Pantelis, 2016). Consistently, a later study by another group (Samamé et al., 2012) reported a small effect size for facial affect recognition and a moderate effect size for ToM in euthymic patients with BD, thus suggesting that ToM is specifically affected in BD.

Overall, the results of the present study suggest that BD patients present altered performances in advanced social cognition, i.e., ToM, that are independent of the mood state and persist during remission and that structural alterations in specific cerebellar portions might be related to such social profiles. Furthermore, the present study allowed us to differentiate the social cognition profiles between BD1 and BD2, highlighting more pronounced alterations in BD2 both in terms of social-behavioral and cerebellar structural patterns. It must be mentioned that just one previous study compared social cognition profiles in remitted bipolar patients types 1 and 2 and showed that both BD1 and BD2 euthymic patients showed impairments in Faux Pas but did not differ in the total Eyes test score (Martino et al., 2011). According to their results, the authors advanced the hypothesis that both automatic (as assessed by the Eyes test) and conscious (as assessed by Faux Pas) components of ToM might rely on different social brain networks (Lee et al., 2004; Sabbagh, 2004), and they might be dissociable. However, it has to be underlined that in the study by Martino et al. (2011), between group difference were only tested for total RMET score while in the present study, we also investigated between-group difference according to the mental states’ valence (positive, negative or neutral; Hudson et al., 2020), and showed a selective alteration when BD patients process stimuli with negative valence. This is also consistent with the evidence that cerebellar recruitment is particularly critical for negative emotions (Ferrucci et al., 2012; Clausi et al., 2022). In conclusion, the social cognition profiles and the pattern of cerebellar correlations differentiating the two groups support at least in part the hypothesis that different social neural networks underlie different ToM components (Lee et al., 2004; Sabbagh, 2004). Future studies specifically investigating and comparing the pattern of FC between cerebral and cerebellar mentalizing regions in BD1 and BD2 may provide further insight into clarifying this issue.

Our investigation has some limitations. The most important limitation is related to the small and unequal sample sizes of the BD1 and BD2 groups. While the present preliminary results need to be confirmed and replicated with larger samples, it must be underlined the novelty and the importance of this first study investigating and comparing the social cognition profile and cerebellar alterations between well-characterized samples of BD1 and BD2 during euthymia. However, despite the limitation of analyzing a small sample size, the consistency with the literature provides support for our conclusions. An important methodological issue needs to be also acknowledged. Indeed, while SPM12 Matlab toolbox is the current version, SPM8 was used to analyze imaging data since past collected data were included in the study. Future studies could re-analyze raw data using SPM12. Another limitation is related to the presence of pharmacotherapy. In spite of previous evidence showing no effect of the pharmacological treatment on the cerebellar structures (Hafeman et al., 2012) there are also studies suggesting that bipolar medication can change the cerebellar GM volume in adult BD patients (Moorhead et al., 2007; Hartberg et al., 2011; Lisy et al., 2011). However, due to the difficulty in finding unmedicated BD patients in remission, at the time of enrolment, all patients were treated. It must be considered that most people with BD need to manage their condition pharmacologically to achieve clinical stability, so studies involving euthymic participants typically recruit people on medications.

Despite these limitations, the exploration and comparison of ToM abilities between BD1 and BD2 patients and the analyses of cerebellar morphological alterations in both cohorts will add a crucial contribution to the comprehension of cerebellar involvement in BD social deficits.

## Data Availability Statement

The raw data supporting the conclusions of this article will be made available by the authors, without undue reservation.

## Ethics Statement

The studies involving human participants were reviewed and approved by IRCCS Santa Lucia Foundation, Rome. The patients/participants provided their written informed consent to participate in this study.

## Author Contributions

Conceptualization: MLu, GO, and MLe. Methodology: GO, MLu, and LS. Formal analysis: GO, MLu, and LS. Investigation: GO, MLu, LS, AG, MS, CP, and MP. Data curation: MLu, GO, LS, and FP. Writing—original draft preparation: GO, MLu, LS, and MLe. Writing—review and editing: GO, MLu, LS, and MLe. Supervision: RD and MLe. All authors contributed to the article and approved the submitted version.

## Funding

The present study was supported by grants from the Italian Ministry of Education, University and Research (MIUR; Grant Number RG120172B8343252) and Department of Psychology, Sapienza University of Rome (Grant Number RM12117A8B3DEE4F) to MLe.

## Footnotes

^1^ https://www.fil.ion.ucl.ac.uk/spm/

^2^ https://www.fmrib.ox.ac.uk/fsl/

## References

[B1] Adams R. B. Jr. Rule N. O. Franklin R. G. Jr. Wang E. Stevenson M. T. Yoshikawa S.. (2010). Cross-cultural reading the mind in the eyes: an fMRI investigation. J. Cogn. Neurosci. 22, 97–108. 10.1162/jocn.2009.21187 19199419

[B2] Amodio D. M. Frith C. D. (2006). Meeting of minds: the medial frontal cortex and social cognition. Nat. Rev. Neurosci. 7, 268–277. 10.1038/nrn1884 16552413

[B3] Aparicio A. Santos J. L. Jiménez-López E. Bagney A. Rodríguez-Jiménez R. Sánchez-Morla E. M. (2017). Emotion processing and psychosocial functioning in euthymic bipolar disorder. Acta Psychiatr. Scand. 135, 339–350. 10.1111/acps.12706 28188631

[B4] Baron-Cohen S. Leslie A. M. Frith U. (1985). Does the autistic child have a “theory of mind”? Cognition 21, 37–46. 10.1016/0010-0277(85)90022-8 2934210

[B5] Baron-Cohen S. Wheelwright S. Skinner R. Martin J. Clubley E. (2001). The autism spectrum quotient (AQ): evidence from Asperger syndrome/high-functioning autism, males and females, scientists and mathematicians. J. Autism Dev. Disord. 31, 5–17. 10.1023/a:1005653411471 11439754

[B187] Bora E. Bartholomeusz C. Pantelis C. (2016). Meta-analysis of Theory of Mind (ToM) impairment in bipolar disorder. Psychol. Med. 46, 253–264. 10.1017/S0033291715001993 26456502

[B7] Bora E. Berk M. (2016). Theory of mind in major depressive disorder: a meta-analysis. J. Affect. Disord. 191, 49–55. 10.1016/j.jad.2015.11.023 26655114

[B184] Bora E. Fornito A. Pantelis C. Yücel M. (2012). Gray matter abnormalities in Major Depressive Disorder: a meta-analysis of voxel based morphometry studies. J. Affect. Disord. 138, 9–18. 10.1016/j.jad.2011.03.049 21511342

[B8] Bora E. Pantelis C. (2016). Social cognition in schizophrenia in comparison to bipolar disorder: a meta-analysis. Schizophr. Res. 175, 72–78. 10.1016/j.schres.2016.04.018 27117677

[B11] Bora E. Vahip S. Gonul A. S. Akdeniz F. Alkan M. Ogut M.. (2005). Evidence for theory of mind deficits in euthymic patients with bipolar disorder. Acta Psychiatr. Scand. 112, 110–116 10.1111/j.1600-0447.2005.00570.x 15992392

[B9] Bora E. Yücel M. Pantelis C. Berk M. (2011). Meta-analytic review of neurocognition in bipolar II disorder. Acta Psychiatr. Scand. 123, 165–174. 10.1111/j.1600-0447.2010.01638.x 21092023

[B10] Bora E. Yucel M. Pantelis C. J. (2009). Cognitive endophenotypes of bipolar disorder: a meta-analysis of neuropsychological deficits in euthymic patients and their first-degree relatives. J. Affect. Disord. 113, 1–20. 10.1016/j.jad.2008.06.009 18684514

[B12] Bourne C. Aydemir Ö. Balanzá-Martínez V. Bora E. Brissos S. Cavanagh J. T. O.. (2013). Neuropsychological testing of cognitive impairment in euthymic bipolar disorder: an individual patient data meta-analysis. Acta Psychiatr. Scand. 128, 149–162. 10.1111/acps.12133 23617548

[B13] Clausi S. Siciliano L. Olivito G. Leggio M. (2022). Cerebellum and emotion in social behavior,” in The Emotional Cerebellum. Advances in Experimental Medicine and Biology, (vol. 1378), eds Adamaszek M. Manto M. Schutter D. J. L. G. (Cham: Springer). 10.1007/978-3-030-99550-8_15 35902475

[B14] Clausi S. Olivito G. Lupo M. Siciliano L. Bozzali M. Leggio M. (2019a). The cerebellar predictions for social interactions: theory of mind abilities in patients with degenerative cerebellar atrophy. Front. Cell Neurosci. 12:510. 10.3389/fncel.2018.00510 30670949 PMC6332472

[B15] Clausi S. Lupo M. Olivito G. Siciliano L. Contento M. P. Aloise F.. (2019b). Depression disorder in patients with cerebellar damage: awareness of the mood state. J. Affect. Disord. 245, 386–393. 10.1016/j.jad.2018.11.029 30423466

[B16] Clausi S. Olivito G. Siciliano L. Lupo M. Bozzali M. Masciullo M.. (2021a). The neurobiological underpinning of the social cognition impairments in patients with spinocerebellar ataxia type 2. Cortex 138, 101–112. 10.1016/j.cortex.2020.12.027 33677324

[B17] Clausi S. Olivito G. Siciliano L. Lupo M. Laghi F. Baiocco R.. (2021b). The cerebellum is linked to theory of mind alterations in autism. A direct clinical and MRI comparison between individuals with autism and cerebellar neurodegenerative pathologies. Autism Res. 14, 2300–2313. 10.1002/aur.2593 34374492 PMC9291804

[B18] D’Agata F. Caroppo P. Baudino B. Caglio M. Croce M. Bergui M.. (2011). The recognition of facial emotions in spinocerebellar ataxia patients. Cerebellum 10, 600–610. 10.1007/s12311-011-0276-z 21503592

[B19] Delvecchio G. Sugranyes G. Frangou S. (2013). Evidence of diagnostic specificity in the neural correlates of facial affect processing in bipolar disorder and schizophrenia: a meta-analysis of functional imaging studies. Psychol. Med. 43, 553–569. 10.1017/S0033291712001432 22874625

[B20] Diedrichsen J. Balsters J. H. Flavell J. Cussans E. Ramnani N. (2009). A probabilistic MR atlas of the human cerebellum. Neuroimage 46, 39–46. 10.1016/j.neuroimage.2009.01.045 19457380

[B21] Fernyhough C. Jones S. R. Whittle C. Waterhouse J. Bentall R. P. (2008). Theory of mind, schizotypy and persecutory ideation in young adults. Cogn. Neuropsychiatry 13, 233–249. 10.1080/13546800801936516 18484289

[B22] Ferrucci R. Giannicola G. Rosa M. Fumagalli M. Boggio P. S. Hallett M.. (2012). Cerebellum and processing of negative facial emotions: cerebellar transcranial DC stimulation specifically enhances the emotional recognition of facial anger and sadness. Cogn. Emot. 26, 786–799. 10.1080/02699931.2011.619520 22077643 PMC4234053

[B23] First M. B. Williams J. B. W. Karg R. S. Spitzer R. L. (2017). SCID-5-CV. Intervista Clinica Strutturata per i Disturbi del DSM-5, Versione per il Clinico; Italiana a cura di Andrea Fossati e Serena Borroni. Milano, Italy: Raffaello Cortina Editore.

[B24] Habel U. Klein M. Kellermann T. Shah N. J. Schneider F. (2005). Same or different? Neural correlates of happy and sad mood in healthy males. Neuroimage 26, 206–214. 10.1016/j.neuroimage.2005.01.014 15862220

[B25] Hafeman D. M. Chang K. D. Garrett A. S. Sanders E. M. Phillips M. L. (2012). Effects of medication on neuroimaging fndings in bipolar disorder: an updated review. Bipolar Disord. 14, 375–410. 10.1111/j.1399-5618.2012.01023.x 22631621

[B26] Hamilton M. (1967). Development of a rating scale for primary depressive illness. Br. J. Soc. Clin. Psychol. 6, 278–296. 10.1111/j.2044-8260.1967.tb00530.x 6080235

[B27] Hartberg C. B. Sundet K. Rimol L. M. Haukvik U. K. Lange E. H. Nesvåg R.. (2011). Subcortical brain volumes relate to neurocognition in schizophrenia and bipolar disorder and healthy controls. Prog. Neuropsychopharmacol. Biol. Psychiatry 35, 1122–1130. 10.1016/j.pnpbp.2011.03.014 21457744

[B28] Harvey P. D. Wingo A. P. Burdick K. E. Baldessarini R. J. (2010). Cognition and disability in bipolar disorder: lessons from schizophrenia research. Bipolar Disord. 12, 364–375. 10.1111/j.1399-5618.2010.00831.x 20636633

[B29] Hein G. Singer T. (2008). I feel how you feel but not always: the empathic brain and its modulation. Curr. Opin. Neurobiol. 18, 153–158. 10.1016/j.conb.2008.07.012 18692571

[B30] Hudson C. C. Shamblaw A. L. Harkness K. L. Sabbagh M. A. (2020). Valence in the reading the mind in the eyes task. Psychol. Assess. 32, 623–634. 10.1037/pas0000818 32237882

[B31] Inoue Y. Yamada K. Kanba S. (2006). Deficit in theory of mind is a risk for relapse of major depression. J. Affect. Disord. 95, 125–127. 10.1016/j.jad.2006.04.018 16797082

[B33] Kerr N. Dunbar R. I. M. Bentall R. P. (2003). Theory of mind deficits in bipolar affective disorder. J. Affect. Disord. 73, 253–259. 10.1016/s0165-0327(02)00008-3 12547294

[B34] Lahera G. Montes J. M. Benito A. Valdivia M. Medina E. Mirapeix I.. (2008). Theory of mind deficit in bipolar disorder: is it related to a previous history of psychotic symptoms? Psychiatry Res. 15, 309–317. 10.1016/j.psychres.2007.08.009 18996602

[B35] Lee K. Farrow T. Spence A. Woodruff P. (2004). Social cognition, brain networks and schizophrenia. Psychol. Med. 34, 391–400. 10.1017/s0033291703001284 15259824

[B36] Lisy M. E. Jarvis K. B. DelBello M. P. Mills N. P. Weber W. A. Fleck D.. (2011). Progressive neurostructural changes in adolescent and adult patients with bipolar disorder. Bipolar Disord. 13, 396–405. 10.1111/j.1399-5618.2011.00927.x 21843279

[B37] Liverta Sempio O. Marchetti A. Lecciso F. (2005). “Faux Pas: traduzione italiana,” in Theory of Mind Research Unit, Department of Psychology. Milan: Catholic University of the Sacred Heart.

[B38] Lupo M. Olivito G. Gragnani A. Saettoni M. Siciliano L. Pancheri C.. (2021). Comparison of cerebellar grey matter alterations in bipolar and cerebellar patients: evidence from voxel-based analysis. Int. J. Mol. Sci. 22:3511. 10.3390/ijms22073511 33805296 PMC8036397

[B39] Lupo M. Olivito G. Siciliano L. Masciullo M. Bozzali M. Molinari M.. (2018a). Development of a psychiatric disorder linked to cerebellar lesions. Cerebellum 17, 438–446. 10.1007/s12311-018-0926-5 29460204

[B40] Lupo M. Olivito G. Siciliano L. Masciullo M. Molinari M. Cercignani M.. (2018b). Evidence of cerebellar involvement in the onset of a manic state. Front. Neurol. 9:774. 10.3389/fneur.2018.00774 30258401 PMC6143664

[B41] Lupo M. Siciliano L. Leggio M. (2019). From cerebellar alterations to mood disorders: a systematic review. Neurosci. Biobehav. Rev. 103, 21–28. 10.1016/j.neubiorev.2019.06.008 31195001

[B42] Mann-Wrobel M. C. Carreno J. T. Dickinson D. (2011). Meta-analysis of neuropsychological functioning in euthymic bipolar disorder: an update and investigation of moderate variables. Bipolar Disord. 13, 334–342 10.1111/j.1399-5618.2011.00935.x 21843273

[B44] Martínez-Arán A. Vieta E. Colom F. Torrent C. Sánchez-Moreno J. Reinares M.. (2004). Cognitive impairment in euthymic bipolar patients: implications for clinical and functional outcome. Bipolar Disord. 6, 224–232. 10.1111/j.1399-5618.2004.00111.x 15117401

[B45] Martino D. J. Strejilevich S. A. Fassi G. Marengo E. Igoa A. (2011). Theory of mind and facial emotion recognition in euthymic bipolar I and bipolar II disorders. Psychiatry Res. 189, 379–384. 10.1016/j.psychres.2011.04.033 21620484

[B46] Martino D. J. Strejilevich S. A. Scápola M. Igoa A. Marengo E. Ais E. D. (2008). Heterogeneity in cognitive functioning among patients with bipolar disorder. J. Affect. Disord. 109, 149–156. 10.1016/j.jad.2007.12.232 18234352

[B47] Meltzoff A. N. Moore M. K. (1989). Imitation in newborn infants: exploring the range of gestures imitated and the underlying mechanisms. Dev. Psychol. 25, 954–962. 10.1037/0012-1649.25.6.954 25147405 PMC4137867

[B48] Merikangas K. R. Akiskal H. S. Angst J. Greenberg P. E. Hirschfeld R. M. Petukhova M.. (2007). Lifetime and 12-month prevalence of bipolar spectrum disorder in the National comorbidity survey replication. Arch. Gen. Psychiatry. 64, 543–552. 10.1001/archpsyc.64.5.543 17485606 PMC1931566

[B49] Mills N. P. Del Bello M. P. Adler C. M. Strakowski S. M. (2005). MRI analysis of cerebellar vermal abnormalities in bipolar disorder. Am. J. Psychiatry 162, 1530–1532. 10.1176/appi.ajp.162.8.1530 16055777

[B50] Moorhead T. W. McKirdy J. Sussmann J. E. Hall J. Lawrie S. M. Johnstone E. C.. (2007). Progressive gray matter loss in patients with bipolar disorder. Biol. Psychiatry 62, 894–900. 10.1016/j.biopsych.2007.03.005 17617385

[B51] Okugawa G. Nobuhara K. Takase K. Kinoshita T. (2007). Cerebellar posterior superior vermis and cognitive cluster scores in drug-naive patients with first-episode schizophrenia. Neuropsychobiology 56, 216–219. 10.1159/000122268 18382120

[B52] Olivito G. Lupo M. Gragnani A. Saettoni M. Siciliano L. Pancheri C.. (2022). Aberrant cerebello-cerebral connectivity in remitted bipolar patients 1 and 2: new insight into understanding the cerebellar role in mania and hypomania. Cerebellum 21, 647–656. 10.1007/s12311-021-01317-9 34432230 PMC9325834

[B53] Olivito G. Serra L. Marra C. Di Domenico C. Caltagirone C. Toniolo S.. (2020). Cerebellar dentate nucleus functional connectivity with cerebral cortex in Alzheimer’s disease and memory: a seed-based approach. Neurobiol. Aging 89, 32–40. 10.1016/j.neurobiolaging.2019.10.026 32081466

[B54] Olivito G. Lupo M. Iacobacci C. Clausi S. Romano S. Masciullo M.. (2018). Structural cerebellar correlates of cognitive functions in spinocerebellar ataxia type 2. J. Neurol. 265, 597–606. 10.1007/s00415-018-8738-6 29356974

[B55] Olley A. L. Malhi G. S. Bachelor J. Cahill C. M. Mitchell P. B. Berk M. (2005). Executive functioning and theory of mind in euthymic bipolar disorder. Bipolar Disord. 7, 43–52. 10.1111/j.1399-5618.2005.00254.x 16225560

[B56] Oztuna D. Elhan A. H. Tuccar E. (2006). Investigation of four different normality tests in terms of type I error rate and power under different distributions. Turkish J. Med. Sci. 36, 171–176.

[B57] Prieto M. L. Cuéllar-Barboza A. B. Bobo W. V. Roger V. L. Bellivier F. Leboyer M.. (2014). Risk of myocardial infarction and stroke in bipolar disorder: a systematic review and exploratory meta-analysis. Acta Psychiatr. Scand. 130, 342–353. 10.1111/acps.12293 24850482 PMC5023016

[B58] Quidé Y. Wilhelmi C. Green M. J. (2020). Structural brain morphometry associated with theory of mind in bipolar disorder and schizophrenia. Psych. J. 9, 234–246. 10.1002/pchj.322 31773921

[B159] Raven J. C. (1949). Progressive matrices. Sets A, Ab, B: Board and book forms. London: Lewis.

[B59] Sabbagh M. (2004). Understanding orbitofrontal contributions to theory of mind reasoning: implications for autism brain and cognition. Brain Cogn. 55, 209–219. 10.1016/j.bandc.2003.04.002 15134854

[B60] Samamé C. Martino D. J. Strejilevich S. A. (2015). An individual task meta-analysis of social cognition in euthymic bipolar disorders. J. Affect. Disord. 173, 146–153. 10.1016/j.jad.2014.10.055 25462409

[B61] Samamé C. Martino D. J. Strejilevich S. A. (2012). Social cognition in euthymic bipolar disorder: systematic review and meta-analytic approach. Acta Psychiatr. Scand. 125, 266–280. 10.1111/j.1600-0447.2011.01808.x 22211280

[B62] Sani G. Chiapponi C. Piras F. Ambrosi E. Simonetti A. Danese E.. (2016). Gray and white matter trajectories in patients with bipolar disorder. Bipolar Disord. 18, 52–62. 10.1111/bdi.12359 26782273

[B63] Schmahmann J. D. Pandya D. N. (1997). The cerebrocerebellar system. Int. Rev. Neurobiol. 41, 31–60. 10.1016/s0074-7742(08)60346-3 9378595

[B64] Schmahmann J. D. Sherman J. C. (1998). The cerebellar cognitive affective syndrome. Brain 121, 561–579. 10.1093/brain/121.4.561 9577385

[B65] Serafin M. Surian L. (2004). “Il test degli Occhi: uno strumento per valutare la ‘teoria della mente”’. Giornale Italiano di Psicologia 31, 213–236. 10.1421/18849

[B66] Sokolov A. A. (2018). The cerebellum in social cognition. Front. Cell. Neurosci. 12:145. 10.3389/fncel.2018.00145

[B67] Sokolovsky N. Cook A. Hunt H. Giunti P. Cipolotti L. (2010). A preliminary characterization of cognition and social cognition in spinocerebellar ataxia types 2, 1 and 7. Behav. Neurol. 23, 17–29. 10.3233/BEN-2010-0270 20714058 PMC5434399

[B68] Stone V. E. Baron-Cohen S. Knight R. T. (1998). Frontal lobe contributions to theory of mind. J. Cogn. Neurosci. 10, 640–656. 10.1162/089892998562942 9802997

[B69] Stoodley C. J. Schmahmann J. D. (2009). Functional topography in the human cerebellum: a meta-analysis of neuroimaging studies. Neuroimage 44, 489–501. 10.1016/j.neuroimage.2008.08.039 18835452

[B70] Torres I. J. Boudreau V. G. Yatham L. N. (2007). Neuropsychological functioning in euthymic bipolar disorder: a meta-analysis. Acta Psychiatr. Scand. Suppl. 434, 17–26. 10.1111/j.1600-0447.2007.01055.x 17688459

[B71] Trouillas P. Takayanagi T. Hallett M. Currier R. D. Subramony S. H. Wessel K.. (1997). International cooperative ataxia rating scale for pharmacological assessment of the cerebellar syndrome. The ataxia neuropharmacology committee of the world federation of neurology. J. Neurol. Sci. 145, 205–211. 10.1016/s0022-510x(96)00231-6 9094050

[B73] Van Overwalle F. Baetens K. Marien M. Vandekerckhove M. (2014). Social cognition and the cerebellum: a meta-analysis of over 350 fMRI studies. Neuroimage 86, 554–572. 10.1016/j.neuroimage.2013.09.033 24076206

[B72] Van Overwalle F. Marien P. (2016). Functional connectivity between the cerebrum and cerebellum in social cognition: a multi-study analysis. Neuroimage 124, 248–255. 10.1016/j.neuroimage.2015.09.001 26348560

[B74] Van Overwalle F. Van de Steen F. Mariën P. (2019). Dynamic causal modeling of the effective connectivity between the cerebrum and cerebellum in social mentalizing across five studies. Cogn. Affect. Behav. Neurosci. 19, 211–223. 10.3758/s13415-018-00659-y 30361864

[B75] Van Overwalle F. Van de Steen F., van Dun, K. Heleven E. (2020). Connectivity between the cerebrum and cerebellum during social and non-social sequencing using dynamic causal modelling. Neuroimage 206:116326. 10.1016/j.neuroimage.2019.116326 31678499

[B76] Vieta E. Suppes T. (2008). Bipolar II disorder: arguments for and against a distinct diagnostic entity. Bipolar Disord. 10, 163–178. 10.1111/j.1399-5618.2007.00561.x 18199235

[B77] Young R. C. Biggs J. T. Ziegler V. E. Meyer D. A. (1978). A rating scale for mania: reliability, validity and sensitivity. Br. J. Psychiatry. 133, 429–435. 10.1192/bjp.133.5.429 728692

